# Disparities in Access to Pediatric Otolaryngology Care During the COVID-19 Pandemic

**DOI:** 10.1177/00034894211048790

**Published:** 2021-10-08

**Authors:** Yvonne Adigwu, Beth Osterbauer, Christian Hochstim

**Affiliations:** 1 University of Southern California—Keck School of Medicine, Los Angeles, CA, USA; 2 Division of Otolaryngology—Head and Neck Surgery, Children’s Hospital Los Angeles, Los Angeles, CA, USA

**Keywords:** vulnerable populations, health services accessibility, pediatric otolaryngology

## Abstract

**Objective:**

Racial/ethnic minority pediatric otolaryngology patients experience health disparities, including barriers to accessing health care. Our hypothesis for this study is that Hispanic or economically disadvantaged patients would represent a larger percentage of missed appointments and report more barriers to receiving care during the COVID-19 pandemic.

**Methods:**

A cross-sectional survey utilizing a modified version of the Barriers to Care Questionnaire was administered via telephone to no-show patients, and median income by zip code was collected. Chi-squared, logistic regression, and Student’s *t*-tests were used to investigate any differences in those who did and did not keep their appointments as well as any differences in mean questionnaire scores.

**Results:**

No-show patients were more likely to be Hispanic than not (OR 2.3, 95% CI: 1.3, 3.9, *P* = .002) and to live in a zip code that had a median income less than 200% of the federal poverty level (OR 1.7, 95% CI: 1.2, 2.4, *P* = .004). Respondents with a high school degree tended to report more barriers to care compared to those with less education.

**Conclusion:**

In our study, we identified ethnic, financial, and logistic concerns that may contribute to patients failing to keep their appointments with the otolaryngology clinic. Future studies are needed to assess the efficacy of measures aimed to reduce these barriers to care such as preventive plans to assist new patients and expanding telehealth services.

## Introduction

Healthcare providers in Los Angeles (LA), California have the privilege of treating a diverse patient population, with 29% of LA. County’s population reported as Hispanic or Latino and 24% of the county’s children living in poverty.^1^ In LA County, a person’s race is strongly associated with their income and socioeconomic status. For example, in 2018, Hispanic residents had a median income of $40 300 compared to $73 900 for White residents of LA County.^2^ Patients belonging to a racial/ethnic minority or low socioeconomic status group generally experience worse health outcomes,^3,4^ and more often utilize hospitals that provide worse experiences for all patients as compared to the hospitals utilized by non-Hispanic White patients.^5^

Pediatric patients are a special group to consider as they lack decision-making capacity regarding their healthcare. Previous research has shown that racial/ethnic minority pediatric patients experience health disparities, including barriers to accessing health care which can lead to worse health outcomes.^3,6^ In the setting of specialty pediatric otolaryngology, racial/ethnic minority pediatric patients or patients from lower socioeconomic households, have been shown to have a higher prevalence of sleep disordered breathing, otitis media, esophageal foreign bodies, and neck abscesses, among other conditions.^7-10^ Furthermore, despite higher prevalence of disease, these patients are less likely to undergo treatments such as adenotonsillectomy or tympanostomy tube insertion.^7,8^ Several factors may contribute to healthcare access and health outcomes among such patients. A study of parents of pediatric otolaryngology patients in a setting of increased financial and geographic barriers to care reported additional barriers related to the general mistrust between this population and the healthcare system.^11^ Insurance coverage can also be an important barrier to children receiving prompt healthcare. In LA County, 47% of children are on Medicaid insurance.^1^ Several studies have demonstrated longer wait times and a lower quality of care for children with publicly provided health insurance.^8,12-14^ Despite recent expansions in government subsidized insurance coverage, barriers to care can persist for pediatric otolaryngology patients, with some publicly insured pediatric patients having been shown to wait a significantly longer time before cochlear implantation than privately insured patients.^15^

In our current study, we sought to investigate potential barriers to accessing pediatric otolaryngology specialty care among our majority Hispanic and publicly insured patient population. A Barriers to Care Questionnaire (BCQ) was previously developed by Seid et al^16^ as an “instrument that conceptualizes barriers to care as a multidimensional construct, as affecting children’s health care at several points in the care process, and as distinct from, yet related to, SES and race/ethnicity.” We utilized a shortened version of the BCQ^16,17^ and investigated the hypothesis that Hispanic or economically disadvantaged patients would represent a larger percentage of missed appointments and report more barriers to receiving care.

## Methods

### Ethics and Study Population

We conducted a cross-sectional telephone survey of patients who failed to keep a scheduled appointment (no-show) with the Children’s Hospital Los Angeles (CHLA) Division of Otolaryngology outpatient clinic between July 1st and August 31st, 2020. In a normal year, this period would be representative of a normal patient load, as the Division does not experience large changes throughout the year, aside from seasonal variations. The CHLA Division of Otolaryngology began conducting in-person, non-emergent visits in June 2020, at 50% capacity, with reduced volume persisting during the study interval. Patients were excluded if they had rescheduled their original missed appointment before contact was attempted. The study was approved by the CHLA Institutional Review Board and verbal consent was obtained over the phone for all study participants. Trained study research associates attempted contact up to 2 times with parents or guardians (caregivers) of no-show patients. Once on the phone, 3 questionnaires were administered to those caregivers that provided verbal consent.

To investigate whether the no-show group was representative of the overall CHLA Otolaryngology clinic population, data was collected from a random sample of 321 patients who kept their appointments during the study period and used for comparison.

### Questionnaires and Data Collection

Three separate questionnaires were administered over the phone to no-show patients that were successfully contacted and provided verbal consent. The first questionnaire consisted of demographic questions, including sex and age of patient, age of the caregiver, and highest completed education of the caregiver. The second questionnaire was a shortened version of the previously validated BCQ.^16,17^ The BCQ is divided into 5 subscales: Skills, Marginalization, Expectations, Knowledge and Beliefs, and Pragmatics. The Skills subscale reflects abilities or acquired strategies to navigate the health care system, while items in Marginalization measure the degree to which negative experiences while receiving past care impact current and future care experiences. Expectation items measure the degree to which caregivers expect poor care, and the Knowledge and Beliefs subscale reflects divergence between what caregivers and doctors believe is best for the child. Pragmatic items assess barriers related to cost and/or logistical issues.^16^ We removed 6 items from the BCQ to make the instrument more relevant to our specific study population, including questions regarding expectations about subsequent visits (many patients are seen as single visit consultations) and feelings about the wider healthcare system that we did not aim to assess. After utilizing our shortened BCQ, we calculated Cronbach’s alpha to assess the reliability, or internal consistency and compared to the original validated BCQ. We found our internal validity comparable (Table 1).

**Table 1.** Comparison of Internal Reliability of Modified Barriers to Care Questionnaire (BCQ), to Original Published Version.

	Cronbach’s from Seid et al^16^	Cronbach’s alpha from modified BCQ
Total	0.95	0.87
Pragmatics	0.85	0.83
Skills	0.86	0.92
Expectations	0.84	0.77
Marginalization	0.91	0.81
Knowledge and beliefs	0.75	0.82

The final questionnaire was an un-validated tool with 4 questions pertaining to barriers associated with the ongoing SARS-CoV2 pandemic, as this was a major factor disrupting care during the study period. As we were in the midst of a pandemic and unsure its course or length, we elected not to pilot these 4 questions. All questionnaires were translated in to Spanish and administered in Spanish over the phone for those caregivers that were Spanish language only.

In addition, data were collected from medical records for all patients including type of appointment (new or follow-up), race, and zip code. Median income by zip code was collected from the American Community Survey^18^ in 2019 adjusted dollars (the most recent data available) and correlated to each participant’s zip code. As in previous studies at this institution,^9,10^ a categorical variable was created as either above or below $51 500, which was 200% of the federal poverty level (FPL) in 2019.

### Statistical Analyses

For each item in the BCQ and COVID questionnaires, caregivers were asked if the item was “no problem” (100), a “small problem” (75), a “problem” (50), a “big problem” (25), or a “very big problem” (0), with higher scores indicating fewer barriers. This scoring system was devised by the authors who first validated the BCQ^16^ and used subsequently by other authors.^11,19^ The mean score was calculated for all questions and by each subscale.

Chi-squared tests and unadjusted logistic regression analyses were used to investigate any differences in those who did and did not keep their appointments, and Student’s *t*-tests to assess any differences in mean BCQ scores. We considered *P*-values ≤.05 to be statistically significant. Study data were collected and managed using REDCap electronic data capture tools hosted at University of Southern California, Keck School of Medicine and analyzed with STATA v13.1 (StataCorp, College Station, TX, USA).^20,21^

## Results

### Population Characteristics

Overall, 211 patients missed a scheduled appointment and were compared to 321 patients who kept their appointments in the same time period. In the caparison of no-show patients to those who kept an appointment, patients were more likely to be a no-show patient if their appointment was a new appointment as opposed to a follow-up appointment (OR 0.5, 95% CI: 0.35, 0.72, *P* < .001, Table 2). In an attempt to further clarify any racial/ethnic differences, we removed all patients listed as “other,” “unknown,” or “missing.” When looking at just this subset of patients with a documented race/ethnicity, no-show patients were more likely to be Hispanic than not (OR 2.3, 95% CI: 1.3, 3.9, *P* = .002, Table 2). No-show patients were also more likely to live in a zip code that had a median income less than 200% of the FPL (OR 1.7, 95% CI: 1.2, 2.4, *P* = .004, Table 2). We then compared these same variables between the no-shows that were unable to be contacted, those who were contacted and declined to participate, and those who completed the survey, and we found no significant differences in appointment type, race/ethnicity or income level, suggesting that the group completing the survey was demographically representative of the overall no-show population (data not shown).

**Table 2.** Comparison of Race/Ethnicity and Socio-Economic Status of Those That Kept an Appointment Versus Those That Did Not Show for Their Appointment.

	No show	Kept appointment	OR	95% CI^a^	*P*-value
	n = 211	n = 321			
Type of appointment					
New	110 (52)	114 (36)	0.5	0.35, 0.72	<.001
Follow up	101 (48)	207 (65)			
Race					
Hispanic	104 (49)	148 (46)	N/A		.008^b^
White	11 (5)	46 (14)			
Asian	6 (3)	16 (5)			
Black	6 (3)	13 (4)			
Other/unknown	84 (40)	98 (31)			
Hispanic versus other^c^	(n = 127)	(n = 223)			
Hispanic	104 (82)	148 (66)	2.3	1.3, 3.9	.002
Other race/ethnicity	23 (18)	75 (34)			
Lives in zip code below 200% FPL^d^	108 (51)	123 (39)	1.7	1.2, 2.4	.004

Odds ratios listed from unadjusted logistic regression with associated confidence intervals and *P*-values.

a Confidence interval.

b From chi-squared test.

c Subset of patients excluding those without a documented race.

d Federal poverty level.

**Figure 1.**
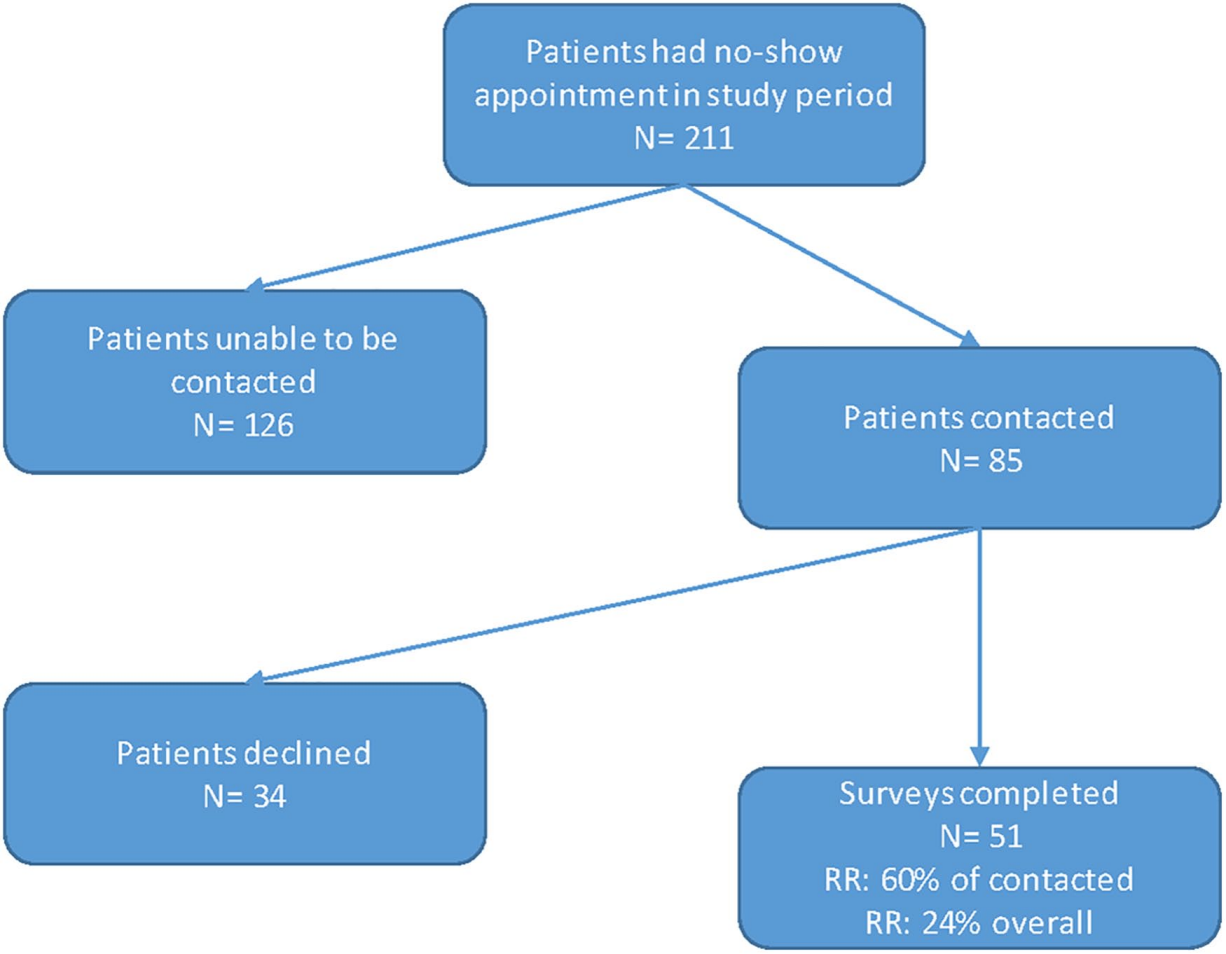
Study flow chart. Abbreviation: RR, response rate.

Of the 211 no-show patients, 85 (40%) were successfully contacted by phone and 51 consented and completed surveys (response rate: 24%, Figure 1). Of the 51 caregivers who completed the survey, 21 (41%) were caregivers to female patients with a mean patient age of 8.9 years (range 9 months-19 years, Table 3). Hispanic patients made up 63% (32/51) of respondents and about half (25/51, 49%) completed the BCQ in Spanish. About 82% (42/51) of responding caregivers were the patient’s mother, 45% (23/51) reporting that they were either married or lived with a partner, and 43% (22/51) had not completed high school (Table 3).

**Table 3.** Overall Characteristics of Barriers to Care Questionnaire respondents.

	All subjects
	N = 51
Female	21 (41)
Age of child in years, mean (SD)	8.9 (5.6)
Lives in zip code below 200% FPL^a^	28 (55)
Hispanic	32 (63)
Language BCQ^b^ conducted	
Spanish	25 (49)
English	26 (51)
Relationship of respondent	
Mother	42 (82)
Father	4 (8)
Other	3 (6)
Marital status	
Married/with a partner	23 (45)
Single/never married	19 (37)
Divorced/widowed/separated	5 (10)
Highest grade completed in school	
Some high school or less	22 (43)
High school diploma/GED^c^	13 (25)
Vocational/Some college	6 (12)
College/graduate	5 (10)

All values presented as n (%) unless otherwise specified.

a Federal poverty level.

b Barriers to Care Questionnaire.

c General Educational Development (high-school equivalency).

### Barriers to Care Questionnaire

**Figure 2.**
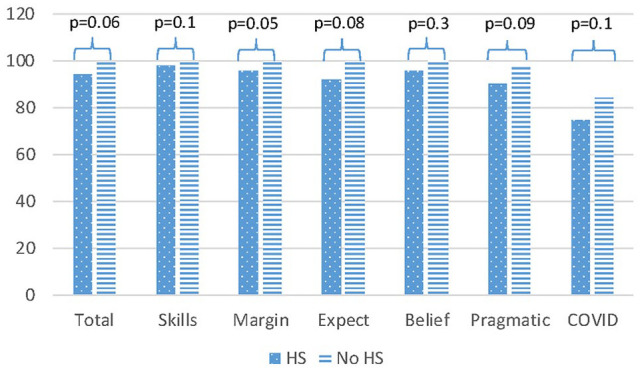
Mean barriers to care questionnaire scores by sub-category, comparison of those with high school degree or higher and those with some high school or less education. Abbreviation: HS, high school. *P*-values listed from paired Student’s *t*-tests.

We found very high BCQ scores overall, equating with few reported barriers. Only 26/51 (51%) of respondents reported any problem. The overall mean score was 96.3 (SD 9.9) and 92.8 (SD 13.0) among those reporting any problem (Table 4). The lowest scoring subcategory overall was Pragmatics (mean score 93.4, SD 15.3, Table 4). In addition, the lowest scoring items, equating with the highest reported barriers were “Having to wait too many days for an appointment” (mean score 81, SD 31.4) and “Getting ahold of the doctor’s office or clinic by telephone” (mean score 94 SD 16.4, Table 4). Next, we evaluated whether the mean scores varied based on other measured characteristics. We found no difference in mean scores based on age of the patient, race, language, income level, or relationship status. We did find some difference based on education level of the caregiver. Respondents with a high school degree or higher level of education had a lower mean score for the Marginalized sub-category (95.7 vs 99.9, *P* = .0496, Figure 2) compared to respondents who did not complete high school. While not reaching the level of significance, the same pattern was observed for the total score, and across all sub-categories, with the higher education group reporting more barriers to care (Figure 2).

**Table 4.** Overall Mean Scores for Each BCQ Questionnaire Item and By Subcategory.

Subcategories of BCQ		Mean BCQ Score (SD)
Overall total score		96.3 (9.9)
Mean score among those reporting any problem (n = 26)		92.8 (13.0)
Skills		98.5 (3.4)
1.	Doctors or nurses not fluent in your language.	100
2.	Doctors or nurses who speak in a way that is too technical or medical.	99.0 (7)
3.	The referral process to our specialty.	97.1 (10.8)
4.	Understanding doctor’s orders.	100
5.	Concerns about insurance coverage.	99.5 (3.5)
6.	Getting enough help with paperwork or forms.	99.0 (7)
7.	Did not understand reason to see the specialist/why they needed to come in	95.1 (18.7)
Marginalization		97.6 (7.5)
8.	Feeling like doctors are trying to give as little service as possible.	94.1 (20.9)
9.	Impatient doctors.	100
10.	Intimidating doctors.	100
11.	Rude office staff.	99.5 (3.5)
12.	Uncaring office staff.	99.5 (3.5)
13.	Getting the doctor to listen to you.	98.5 (10.6)
14.	Getting your questions answered.	98.5 (10.6)
15.	Being judged on your appearance, your ancestry, or your accent.	100
Expectations		95.4 (16.3)
16.	Doctors rushing you and your child through the visit.	100
17.	Offices and staff that are not child-friendly.	100
18.	Mistakes made by doctors or nurses.	98.5 (10.6)
19.	Worrying that doctors and nurses will not do what is right for your child	99 (7)
20.	Doctors treating the symptom without finding out the cause of the illness.	98 (11.1)
21.	Getting a thorough examination.	95 (20.8)
22.	Lack of communication between my child’s doctor and the ENT specialist.	95.4 (20.2)
Knowledge and beliefs		97.5 (14.2)
23.	Disagreeing with the doctor’s orders.	98.5 (10.6)
24.	Doctors not believing in home or traditional remedies.	99.5 (3.5)
25.	Doctors giving you instructions that seem wrong.	100
26.	Doctors or nurses that have different ideas about health than you do.	100
Pragmatics		93.4 (15.3)
27.	Getting to the doctor’s office.	96.5 (15.1)
28.	Getting ahold of the doctor’s office or clinic by telephone.	94 (16.4)
29.	Having to wait too many days for an appointment.	81 (31.4)
30.	Getting care after hours or on the weekends.	96.5 (11.3)
31.	Having to take care of household responsibilities.	99.5 (3.5)
32.	Having to take time off work.	96 (14.6)
33.	Having to wait too long in the waiting room.	98 (8.5)
34.	Meeting the needs of other family members.	98.5 (11.6)
35.	The cost of health care.	98.5 (10.6)

### COVID Questionnaire

Of the 51 respondents, 31 (61%) reported any problem with access to care due to the COVID pandemic. The mean score for this separate subscale was 79.0 (SD 22.9) and among only those reporting any problem, the mean score was much lower at 65.5 (SD 19.8, Table 5). While this was expected, it was a much lower score than those reporting any problem on the main BCQ (mean 92.8, SD 13.0, Table 4). The lowest scoring item in the COVID questionnaire was “Avoiding doctor’s office due to the coronavirus” with a mean score of 71.2 (SD 41.6, Table 5).

**Table 5.** COVID Questionnaire Scores.

		Mean score (SD)
COVID		79.0 (22.9)
Mean score among those reporting any problem (n = 31)		65.5 (19.8)
1.	Office closed due to the coronavirus/COVID	92 (20.5)
2.	Appointments were cancelled by the doctor’s office due to coronavirus	84 (29.8)
3.	Avoiding doctor’s office due to the coronavirus	71.2 (41.6)
4.	Avoiding public transportation due to the coronavirus	74 (38.4)

## Discussion

To our knowledge, this is the first study attempting to characterize the barriers to pediatric otolaryngology care among patients who fail to keep their appointments. We found that no-show patients were more likely to be new patients to the clinic, Hispanic, and to come from households living in zip codes with a median income below 200% of the FPL. Among this group of no-show patients, those caregivers who reported higher levels of completed education also reported more barriers to care, somewhat counter to our original hypothesis. In a similar study by Yang et al^15^ of pediatric patients with cochlear implants, a larger percentage of privately insured patients reported barriers to care compared to publicly insured patients. The authors hypothesized that this difference could be due in part to a difference in baseline expectations, which we believe could potentially explain the observed difference in our population as well.

In a recent community health needs assessment conducted by CHLA,^1^ 60.5% of respondents reported that access to health care was a top health care concern or issue. Moreover, among the barriers noted in the health needs assessment to achieving access, financial concerns and insurance coverage were the top 2. Our findings from the demographic analyses echo this pattern with households living in zip codes with a median income below 200% of the FPL more likely to miss scheduled appointments. Somewhat similarly, among the group of BCQ respondents in our study, one of the lowest scoring sub-categories was Pragmatics, which assessed barriers related to cost and/or logistical issues. Previous studies utilizing the BCQ in different settings also found Pragmatics to be the sub-category with the most reported problems. In a study of caregivers of cleft lip/cleft palate children in Michigan, Bennet et al^19^ reported a mean BCQ score of 91.5 for Pragmatics. Razdan et al^11^ found a mean Pragmatics score of 90.8 in rural West Virginia, and in a study from a children’s hospital in Washington DC, Pragmatics was the sub-category with the largest percentage of patients reporting any problem.^15^

As we chose to shorten the previously validated BCQ, direct comparisons are not strictly possible. However, we did notice similar patterns to previous reports in that we found lower rates of reported barriers to care than were expected. As the patient population we surveyed had all missed an appointment, we hypothesized that we may uncover more barriers to care than previous reports that surveyed patients who had already arrived in clinic.^11,15^ Several factors could have contributed to this our observed low rates of reported barriers, yet it is difficult to reach any conclusions on this topic without also surveying patients who kept their appointments for comparison. However, the authors’ demographic analyses comparing no-shows to those who kept an appointment revealed disparities of particular interest.

A major consideration for the current study was that it was conducted in the midst of a global pandemic, a time when most people’s access to health care has been impacted. Our hypothesis was that the pandemic might have a disproportionate impact on disadvantaged groups in terms of access to care. We created COVID-specific questions to help address the impact of the pandemic on access to care in our population, and indeed this was the area with the lowest scores overall, equating to the most reported problems. It will be important for future studies to examine barriers to care in the post-pandemic environment going forward.

Other limitations of the current study include the small number of respondents and a potential response bias. Although low, our response rate of 24% is comparable to other published response rates between 22% and 35% for telephone surveys specifically.^22-24^ It may be that respondents who agree to participate are a group that perceive fewer barriers to care overall. Although we compared those who responded, declined and were unable to be reached, these groups could differ from each other in ways that we were unable to measure. In addition, the BCQ was administered over the phone potentially introducing selection and recall biases. Respondents may be less apt to remember why they missed an appointment, or report problems directly to a person, as opposed to completing a survey alone, either paper or web based. However, the target population of patients who have already missed an appointment are inherently difficult to contact, therefore future studies should devise more inclusive ways to reach and survey such participants.

## Conclusion

Access to health care is a major concern for residents of LA County, with an overall health access rating near the bottom (45th out of 57 counties) for the state of California.^1^ In both our demographic and survey analyses, we identified ethnic, financial, and logistic concerns that may contribute to patients failing to keep their appointments with the otolaryngology clinic. Preventive plans to assist new patients and expanding telehealth services are strategies to investigate as they may contribute to reducing these barriers to care. Future studies should focus on larger sample sizes in order to capture a more heterogeneous group of patients.
